# Influence of Acid Adaptation on the Probability of Germination of *Clostridium sporogenes* Spores Against pH, NaCl and Time

**DOI:** 10.3390/foods9020127

**Published:** 2020-01-24

**Authors:** Antonio Valero, Elena Olague, Eduardo Medina-Pradas, Antonio Garrido-Fernández, Verónica Romero-Gil, María Jesús Cantalejo, Rosa María García-Gimeno, Fernando Pérez-Rodríguez, Guiomar Denisse Posada-Izquierdo, Francisco Noé Arroyo-López

**Affiliations:** 1 Department of Food Science and Technology, Agrifood Campus of International Excellence, Universidad de Córdoba, 14014 Córdoba, Spain; bt1gagir@uco.es (R.M.G.-G.); b42perof@uco.es (F.P.-R.); 2 Department of Food Technology, Public University of Navarra, Campus de Arrosadia, E-31006 Pamplona, Spain; olagueramos@gmail.com (E.O.); iosune.cantalejo@unavarra.es (M.J.C.); 3 Food Biotechnology Department, Instituto de la Grasa (IG-CSIC), University Campus Pablo de Olavide, Building 46, Ctra. Utrera, km 1, 41013 Seville, Spain; emedina@ig.csic.es (E.M.-P.); garfer@cica.es (A.G.-F.); fnoe@ig.csic.es (F.N.A.-L.); 4 Technological Applications for Improvement of the Quality and Safety in Foods, R&D Division, Crta. Marbella 22. Guaro, 29108 Málaga, Spain; v.romero@oleica.es

**Keywords:** *Clostridium*, logistic regression, acid-adapted strains, predictive models, table olives, fermented vegetables

## Abstract

The *Clostridium* sp. is a large group of spore-forming, facultative or strictly anaerobic, Gram-positive bacteria that can produce food poisoning. The table olive industry is demanding alternative formulations to respond to market demand for the reduction of acidity and salt contents in final products. while maintaining the appearance of freshness of fruits. In this work, logistic regression models for non-adapted and acid-adapted *Clostridium* sp. strains were developed in laboratory medium to study the influence of pH, NaCl (%) and time on the probability of germination of their spores. A *Clostridium* *sporogenes* cocktail was not able to germinate at pH < 5.0, although the adaptation of the strains produced an increase in the probability of germination at 5.0–5.5 pH levels and 6% NaCl concentration. At acidic pH values (5.0), the adapted strains germinated after 10 days of incubation, while those which were non-adapted required 15 days. At pH 5.75 and with 4% NaCl, germination of the adapted strains took place before 7 days, while several replicates of the non-adapted strains did not germinate after 42 days of storage. The model was validated in natural green olive brines with good results (>81.7% correct prediction cases). The information will be useful for the industry and administration to assess the safety risk in the formulation of new processing conditions in table olives and other fermented vegetables.

## 1. Introduction

The *Clostridium* sp. is a foodborne pathogen that may be present in a wide variety of low acid fermented foods, being able to produce illness after its ingestion. Though there are some species for commercial use in foods such as *C. acetobutylicum*, most of them are considered spoilage or pathogenic bacteria for humans like *C. perfringens*, *C. botulinum*, *C. butyricum*, *C. tetani*, *C. difficile*, and *C. sordellii* [1].

Their spores are ubiquitously present in warm-blooded animals, and distributed in the environment, soil and water, so that they can contaminate foods during processing. In the case of *C. botulinum*, it can produce highly toxic neurotoxins, causing potentially fatal human diseases after the germination and growing of vegetative cells [2]. Therefore, to avoid the production of neurotoxin, it is essential to prevent its germination. The toxin types are classified as A, B, C, D, E, F and G. Human botulism has been mainly described with the strains of *C. botulinum* that produce toxin types A, B and E. Botulism outbreaks caused by different home-prepared or preserved foods have been widely reported in the literature, most of them due to improperly pasteurized or packed home-canned vegetables [3,4]; home-made oil condiments and sauces [5,6,7]; fishery products [8,9]; cheese [10,11]; or meat products [12,13]. Although it is not usual, several outbreaks in table olives have also been reported to be mainly associated with black table olives [14]. According to the last report on the trends and sources of zoonoses, zoonotic agents and food-borne outbreaks in the European Union (EU) in 2017, five strong-evidence outbreaks and 26 human cases associated with botulism were reported. Botulism cases involved hospitalization rates higher than 50%, *C. botulinum* being reported as the agent with one of the highest fatality rates [15].

The food industry requires alternative formulations with reduced acidity and salt content for canned or fermented vegetable foods, given the increasing demand by consumers for healthier and more convenient foods. However, the changes could represent a risk for the population. In the specific case of the table olive industry, salt reduction below 6% is necessary to respond to market demand. Also, excessively low pH can affect the green appearance of fruits by the degradation of chlorophylls into pheophytins. Thus, research has been oriented to study the influence of these environmental factors on the survival, growth and toxin production of *Clostridium* sp., to assess the risk associated with new packaging conditions. Temperature, pH and Knack, together with a combination of different preservatives (sodium lactate, sorbic acid, lysozyme or nisin), have been widely studied in culture media and different commodities [16,17,18,19,20], with most of them focused upon the growth and germination probability in different formulations, or after thermal processing.

Food safety assurance in canned or fermented, acidic foods from vegetable origins (olives, tomatoes, pickles, etc.) is typically achieved by lowering the pH below 4.6 (acidic) to prevent the proliferation of the *Clostridium* strains. However, this limit could be compromised by acid-adapted strains that could persist in contaminated products, provided the pH levels and oxygen conditions allow their germination and the subsequent production of neurotoxins. Furthermore, the survival ability of *C. botulinum* to grow, and the production of toxin in acidic environments (pH < 4.6) has been described in earlier studies, performed in both culture media and food model matrices [21,22,23,24]. Such ability can be partly explained by the implementation of a pH-inducible acid tolerance response (ATR) in sporulated bacteria at acidic pH (5.0). This tolerance produces a remarkable cell elongation [25], and increases resistance to stress at sublethal growth conditions. More recent works have studied the role of the cold shock protein-coding genes (*csp*), which are involved in growth at low temperature. Specifically, strains of *C. botulinum* having the genes *cpsB* or *cspC* develop adaptation mechanisms against NaCl, pH and ethanol stresses [26].

Therefore, a better understanding of the microbial behavior (germination and toxin production) of acid-adapted *Clostridium* sp. against environmental conditions is of particular interest for food safety assurance in the table olive industry. To this aim, microbial predictive models in foods can be effectively applied by scientists, food operators, public administration and governmental authorities, to maintain microbial quality and ensure safety [27]. 

The development of the probability models of *Clostridium* sp. could be useful to estimate the possibility of germination and toxin production at low infection doses, thus assisting manufacturers in the decision-making process for food quality and safety assurance [28]. Previous works have been oriented to establish food formulations for nonthermal preservation treatments by using inhibitory factors and their interactions to assess the *C. botulinum* growth probability [18,19,20,21,29,30]. Nevertheless, dedicated probability models using acid-adapted strains of *Clostridium* sp. have not been found in the literature.

In this work, logistic regression models for non-adapted and acid-adapted *Clostridium sporogenes* strains to study the influence of pH, NaCl and incubation time on the probability of germination of their spores, were developed. The factor ranges have been selected so that the model could be applied to table olive processing.

## 2. Materials and Methods

### 2.1. Strains and Culture Conditions

In the present study, *C. sporogenes* strains were used as a non-toxigenic equivalent of proteolytic *C. botulinum*, since it also causes food spoilage [16]. Thus, its use is highly recommended in challenge test studies, as microbial responses can be extrapolated to *C. botulinum* behavior [31]. Three strains of *C. sporogenes* were obtained from the Spanish Type Culture Collection (CECT), namely CECT 485 (ATCC 19404; NCTC 532); CECT 892 (ATCC 3584; NCIMB 10696); and CECT 4990 (CIP 79.39). Freeze-dried pellets were resuscitated using a small volume (2–3 mL) of Liver Broth (LB, Oxoid, Basingstoke, Hampshire, England, UK) incubating at 37 °C overnight. The use of cooked meat media for the resuscitation of sporulated bacteria has been suggested by other authors [16]. Frozen stock cultures were maintained in LB at −80 °C in Eppendorf tubes with 15% glycerol until use. Activation of vegetative cells was carried out by transferring 0.1 mL to 10 mL screw-cap tubes of LB, incubating at the same conditions above. Then, 0.1 mL of LB was transferred to 10 mL screw-cap tubes of Fluid Thioglycollate Medium (FTM, Oxoid, Basingstoke, Hampshire, England, UK), incubating at 37 °C for 48 h, until the medium became turbid. Enumeration of colonies was done by pouring 1 mL into tryptose sulphite cycloserine (TSC) agar with D-cycloserine (Oxoid, Basingstoke, Hampshire, England, UK) incubating in anaerobic jars at 37 °C for 48–72 h. Enumeration was also done using a Thoma cell counting chamber (Hirschmann Instruments™ 8100103) where the morphology of the cells and spores were visualized. 

### 2.2. Preparation of Inoculum

To obtain the suspension of spores, 1 mL of vegetative cells in FTM was transferred to sterile 100 mL flasks of FTM to produce more vegetative cells incubating at 37 °C for 48 h. Flasks were then stored at room temperature to enhance sporulation. Sporulation was daily checked by Gram staining together with enumeration in a Thoma cell counting chamber, and spores were harvested after one week, where a high population of spores (80%) was achieved [16]. Before inoculation, the FTM flasks were heated at 87 °C for 15 min to activate sporulation and to destroy vegetative cells. Sporulated cultures were washed twice with phosphate-buffered saline (PBS) (Medicago AB, Uppsala, Sweden) by centrifugation at 4100 rpm (Jouan C4 i, Thermo Electron Corporation, France) for 10 min and finally re-suspended in saline solution (0.85%). The cocktail of *C. sporogenes* spores was prepared by mixing volumes of 4 mL from each strain suspension (1 × 10^9^ spores/mL approx.) in a sterile test tube.

### 2.3. Acid Adaptation of C. sporogenes Strains

From the 10 mL tubes of FTM, the gradual acid adaptation of *C. sporogenes* strains was achieved by using decreasing pH levels (6.5, 6.0, 5.75 and 5.5). The pH of each prepared broth was aseptically adjusted with hydrochloric acid (1M). One ml of the grown culture at pH 7.2 (alkali) was transferred to a sterile tube of FTM at pH 6.0, incubating at 37 °C in anaerobic jars until visible turbidity was observed. Further, the same transfer was repeated to the subsequent tubes at the decreasing pH levels. 

When an acid-adapted culture was obtained at pH 5.5, 1 mL of vegetative cells in FTM was transferred into sterile 100 mL flasks of FTM with their pH adjusted to 5.5. Inoculum preparation of acid-adapted spores was then carried out as explained above, using modified pH media.

### 2.4. Experimental Design

A full factorial design, including 32 combinations of eight pH and four NaCl levels, was achieved for non-adapted and acid-adapted strains (pre-incubated at pH 5.5). The influence of pH (4.0, 4.5, 5.0, 5.5, 5.75, 6.0 and 7.0) and NaCl (0%, 2%, 4% and 6%) was assessed. The sodium chloride (NaCl) percentage was calculated considering the salt content of the initial Differential Reinforced Clostridial Medium (DRCM, Oxoid, Basingstoke, Hampshire, England, UK) (0.5%). The pH was measured with a pH/mv-meter digit 501 (Crison, Barcelona, Spain), and its adjustment was aseptically performed using hydrochloric acid (HCl) (1M). Once modified, all media were sterilized, and subsequently, the NaCl concentrations and pH values were verified. Temperature was not initially considered as a model variable, so that it was assumed that table olives can be eventually stored at relatively high temperature conditions during summer periods. Microbial responses were recorded daily for 42-days incubation, so a total of 1344 growth/no growth data (32 pH and NaCl combinations × 42 time points) were obtained for the development of the logistic regression models. 

### 2.5. Inoculation Procedure and Germination Assessment

For assessing the germination probability of the *C. sporogenes* cocktail, for each physiological state condition (non-adapted and acid-adapted), DRCM was used. In this culture, medium *Clostridia* can reduce sulphite to sulfide—forming iron sulfide. Iron (III) citrate is included in the formulation as an indicator of sulphite reduction. For assessing germination, microtiter plates of 10 × 10 wells each were inoculated with 300 µL medium + 100 µL of inoculum. Eight wells per condition were inoculated with two blanks (400 µL of uninoculated DRCM). Appropriate dilutions of the initial inoculum were made in such a way that a concentration of 1 × 10^6^ spores/well was reached. Afterwards, microtiter plates were covered with a lid and sealed with paraffin. Incubation was done in 2.5 L anaerobic jars using AnaeroGen™ sachets (Thermo Scientific) for the gas generation, so that oxygen concentration was reduced below 1% and CO_2_ reached 9%–13% [32]. Then, the anaerobic jars were tightly closed and incubated at 30 °C for 42 d. Germination was visually recorded daily when the medium darkened, indicating that the sulphite reduction had occurred and iron sulfide had been formed. At the end of the experiments, the microbial concentration of *C. sporogenes* was confirmed by pour plating the volume of the well (400 µL) onto Sodium polyanethol sulfonate (SPS) and tryptose sulphite cycloserine (TSC) (Oxoid, Basingstoke, Hampshire, England, UK). Positive germination was verified if there was a 1-log increase in the microbial concentration with respect to the inoculation moment. Contaminated and turbid wells which did not show blackening were discarded.

### 2.6. Development of Logistic Regression Models

The whole dataset was implemented in an Excel spreadsheet, and a polynomial logistic regression equation was fitted to the model data observed. Generally, this type of model contains a right-hand side term (which is a polynomial equation) and a left-hand side term, named “logit *p*”, $logit\;p=ln\left(\frac{p}{1-p}\right)$ [33]. The equation used in this study was a second-order linear logistic regression model, as follows:

(1) $$\begin{array}{ll} Logit\;p= & ln\left(\frac{p}{1-p}\right)\\ & \;=b_{0}+b_{1}*time+b_{2}*pH+b_{3}*NaCl+b_{4}*time*NaCl+b_{5}\\ & \;*NaCl*pH+b_{6}*time*pH+b_{7}*time*NaCl*pH+b_{8}*pH^{2}+b_{9}\\ & \;*NaCl^{2}+b_{10}*time^{2} \end{array}$$

where *p* is the probability of germination, and *b*_0_–*b*_10_ are the coefficients to be estimated. Time units were set in days.

From the observed conditions, a dataset was selected for model development (training), and internal validation was made using conditions within the model range domain. Conditions selected are represented in Table 1. The logistic regression models were fitted in R v3.4.0 (R Project for Statistical Computing) by using the *glm* function. A forward stepwise process was used by adding the significant variables (*p* < 0.05) at each step. With this procedure, a biologically consistent model was obtained, in accordance with the data observed. For assessing predictions, the cut-off value was established at 0.125, thus considering that germination was produced if there were at least 1 out of 8 positive wells.

### 2.7. Assessment of Model’s Performance

Once the model was obtained, its performance was evaluated using the goodness of fit statistics and predictive performance indices, which was determined by (i) the likelihood ratio test (−2*lnL*), where L is the likelihood at its optimum; (ii) Akaike Information Criterion (*AIC* = −2*lnL* + 2*k*, where *k* is the number of parameters in the model); (iii) the determination coefficient (R^2^-Nagelkerke), which quantifies the proportion of variation explained by the logistic regression model; and iv) the Hosmer–Lemeshow (HL) statistic. The −2*lnL* and the AIC can be used to rank models based on the same dataset, where lower values indicate better fitting models. The HL statistic indicates if the model fits the data adequately. This statistic divides the number of times in which growth occurred (observed events) into approximately ten groups (based on the predicted probabilities), and then, compares the observed and the expected number of events in the groups through a contingency table by using the Pearson coefficient. Lower values of the HL statistic indicate a better fit. The area under the Receiver Operating Characteristic (ROC) curve, c, is a measure of discrimination, obtained from a plot sensitivity (the proportion of observed events that were correctly predicted to be events), against the complement of specificity (the proportion of observed non-events that were correctly predicted to be non-events). The closer the value of c is to 1, the higher is the discrimination. For a better illustration of the adjustment of the developed model to the data observed, predicted germination probabilities at 0.125, 0.5 and 0.9 were calculated maintaining constant the pH and NaCl terms, and then were plotted in contour graphs. 

### 2.8. Validation of the Logistic Regression Models in Table Olive Brines

The logistic regression models for the non-adapted and acid-adapted *C. sporogenes* strains were validated in brines from fermented table olives. Brines were obtained from directly brined green Aloreña fermentations. First, brines were centrifuged at 4000 rpm for 10 min, and the supernatant was filter-sterilized using a bacteriological filter with a pore size of 0.22 μm Ø, (Millipore filter Unit-Express plusPES, Billerica, MA, USA). Then, sterilized brines were adjusted to the different pH, and the NaCl conditions explained in Section 2.4. The studied combinations included three pH levels (5, 5.5 and 6) and two NaCl concentrations (4% and 6%). Afterwards, the adjusted brines were aseptically transferred into 7 mL sterile, screw-cap tubes and inoculated with 0.1 mL of a spore suspension of the *C. sporogenes* cocktail. Finally, the tubes were incubated anaerobically at 30 °C for 13 d. Germination of *C. sporogenes* was daily assessed through plate counting in TSC and SPS agars.

## 3. Results

### 3.1. Performance of the Logistic Regression Models

Logistic regression models were developed for estimating the probability of germination of non-adapted and acid-adapted *C. sporogenes* strains. Estimation of the significant coefficients together with their corresponding standard errors and *P*-values, are represented in Table 2. It should be remarked that for the pH, the ln-transformed term was used for the logistic model of non-adapted strains for improving accuracy.

It should be noticed that the linear pH term was not significant (*P* > 0.05) for the model of the non-adapted strains. The performance statistics obtained indicate reasonable goodness of fit of the models obtained, mainly due to the high values of R^2^-Nagelkerke (>0.921) and AIC values (Table 3). The HL statistics gave *P*-values higher than 0.05 for both models, thus indicating a good adjustment to the observed data. These values are in line with other logistic models published in the literature [18,19,34]. However, a higher degree of accuracy was obtained for the logistic model of acid-adapted strains, given the lower values of AIC and log-likelihood in comparison to that of non-adapted strains.

Through the calculation of the area under the ROC curve, the corrected classified cases were calculated for model and validation data. Their percentages were estimated considering a cut-off value of 0.125 for the probability of germination (≥1/8 germinated wells). The classification percentages of observed vs. predicted conditions are shown in Table 4. The logistic models provide a certain margin of safety, since most of the misclassified cases were considered as fail-safe (i.e., germination was predicted, while no germination was observed). These findings can be translated positively into an industrial context, since by using the model, safe formulations can be designed in such a way that the germination of *C. sporogenes* is prevented.

According to the proportion of correctly classified cases (Table 4), for the acid-adapted strains, 32 training and 12 validation conditions were misclassified as fail-safe, while only one case was considered fail-dangerous for the training dataset. For the validation dataset, all misclassified cases were fail-safe. Regarding the logistic model for the non-adapted strains, all deviations were fail-safe. For the training and validation datasets, 55 and 26 cases were misclassified, respectively. However, the average proportion of correctly classified cases was higher than 92%.

### 3.2. Effect of Environmental Factor on the Probability of Germination of Non-Adapted and Acid-Adapted C. sporogenes Strains

**Figure 1:**
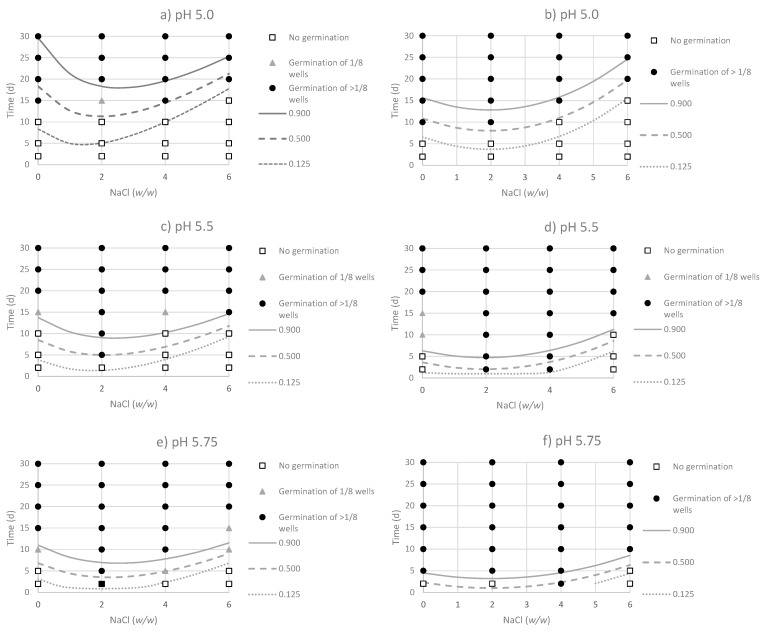
Contour plots for the observed germination responses and predicted probabilities (*p* = 0.125, *p* = 0.500 and *p* = 0.900) for the non-adapted (panels **a**,**c**,**e**) and acid-adapted strains (panels **b**,**d**,**f**) of *C. sporogenes* at pH levels 5.0, 5.5 and 5.75.

**Figure 2:**
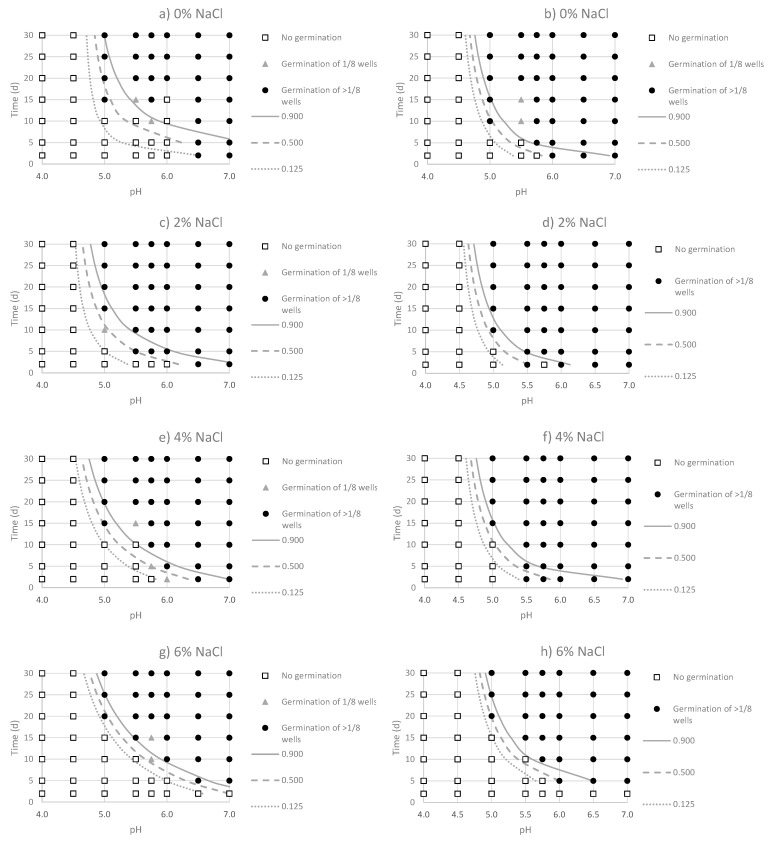
Contour plots for the observed germination responses and predicted probabilities (*p* = 0.125, *p* = 0.500 and *p* = 0.900) for the non-adapted (panels **a**,**c**,**e**,**g**) and acid-adapted strains (panels **b**,**d**,**f**,**h**) of *C. sporogenes* at NaCl concentrations of 0%, 2%, 4% and 6% *w*/*w*.

The observed responses confirmed the high sensitivity of *C. sporogenes* to low pH values, since the microorganism was not able to germinate at pH < 5.0 at any tested condition. Overall, the acid-adaptation of the strains produced a faster germination of spores at close to the limiting conditions of the pH and NaCl levels, as observed at moderately acidic pH (5.0–5.5) combined with a high (>4%) NaCl concentration. The main advantages of logistic regression models are that they can set the level of stringency required at certain environmental conditions. Contour plots representing the germination responses of both non-adapted and acid-adapted strains of *C. sporogenes* as a function of pH (5.0, 5.5 and 5.75) and incubation time (0–30 days), is shown in Figure 1. Lines of constant probabilities were then compared graphically with the experimental data at values of *p* = 0.125, *p* = 0.500 and *p* = 0.900. The homologous germination responses at NaCl concentrations of 0%, 2%, 4% and 6% as a function of pH and incubation time (0–30 days), are represented in Figure 2. A narrower transition between germination and non-germination boundaries was obtained for the acid-adapted strains. This result indicates that small changes in pH and NaCl formulations can govern the germination responses of acid-adapted *C. sporogenes* strains. The more abrupt germination/no-germination transition in the case of the acid-adapted strains produced fewer intermediate conditions, where binary responses were observed. These combinations are represented in Table 5. For non-adapted strains, intermediate conditions were observed at pH 5, 5.5 and 5.75. For the acid-adapted strains, these conditions were mainly observed at pH 5.0 and 5.5. All binary responses implied positive germination in > 1/8 wells, so that all of this model’s predictions yielded probabilities higher than 0.125.

The results showed that at acidic pH values (5.0), the acid-adapted strains germinated at 10 days’ incubation at NaCl concentrations ≤ 2% (Figure 1b), while the germination time of the non-adapted strains increased until 15 days at these NaCl concentrations (Figure 1a). According to the model’s predictions, the growth boundary is set at pH 5.0, NaCl 3.95% and 10 d incubation for non-adapted strains, while this boundary is shifted to an increased NaCl concentration of 4.92% (pH 5 for a 10-days incubation) for the acid-adapted strains.

As expected, as pH increases, higher NaCl concentrations are required to prevent germination. At pH 5.5, germination of non-adapted strains occurred after 15-days incubation, regardless of NaCl concentration (Figure 1c). For acid-adapted strains, the presence of NaCl activated the germination of spores, since at concentrations ≥ 2%, it occurred after 24 h incubation at pH 5.5 (Figure 1d). Using pH 5.5 and 6% NaCl, the models predicted germination after 9.26 and 6.20 days of incubation for the non-adapted and acid-adapted strains, respectively.

At pH 5.0, NaCl concentrations ≥ 4% delayed germination for more than 10 days (Figure 1a,b). However, by increasing the pH to 5.5, both 2% and 4% NaCl concentrations produced germination in 24 h for acid-adapted strains, while non-adapted ones delayed germination after the 15^th^ day of incubation (Figure 1c,d).

NaCl concentrations of 2% and 4% produced a faster germination of *C. sporogenes* in comparison to conditions in the absence of NaCl and pH levels ≤ 6.0. For instance, in the case of the non-adapted strains, germination was produced, in the absence of NaCl, after 20 days of incubation at pH 6.0 (Figure 2a), but the pathogen was germinated on the 5th day in the presence of 2% NaCl (Figure 2c). For the acid-adapted strains, germination was produced at more limiting conditions, since at pH 5.5 and 2% and 4%, germination was observed after 24 h incubation (Figure 2d,f). However, when NaCl was not added, germination of all wells took place ≥ 20 days at pH 5.5 (Figure 2b). 

**Figure 3:**
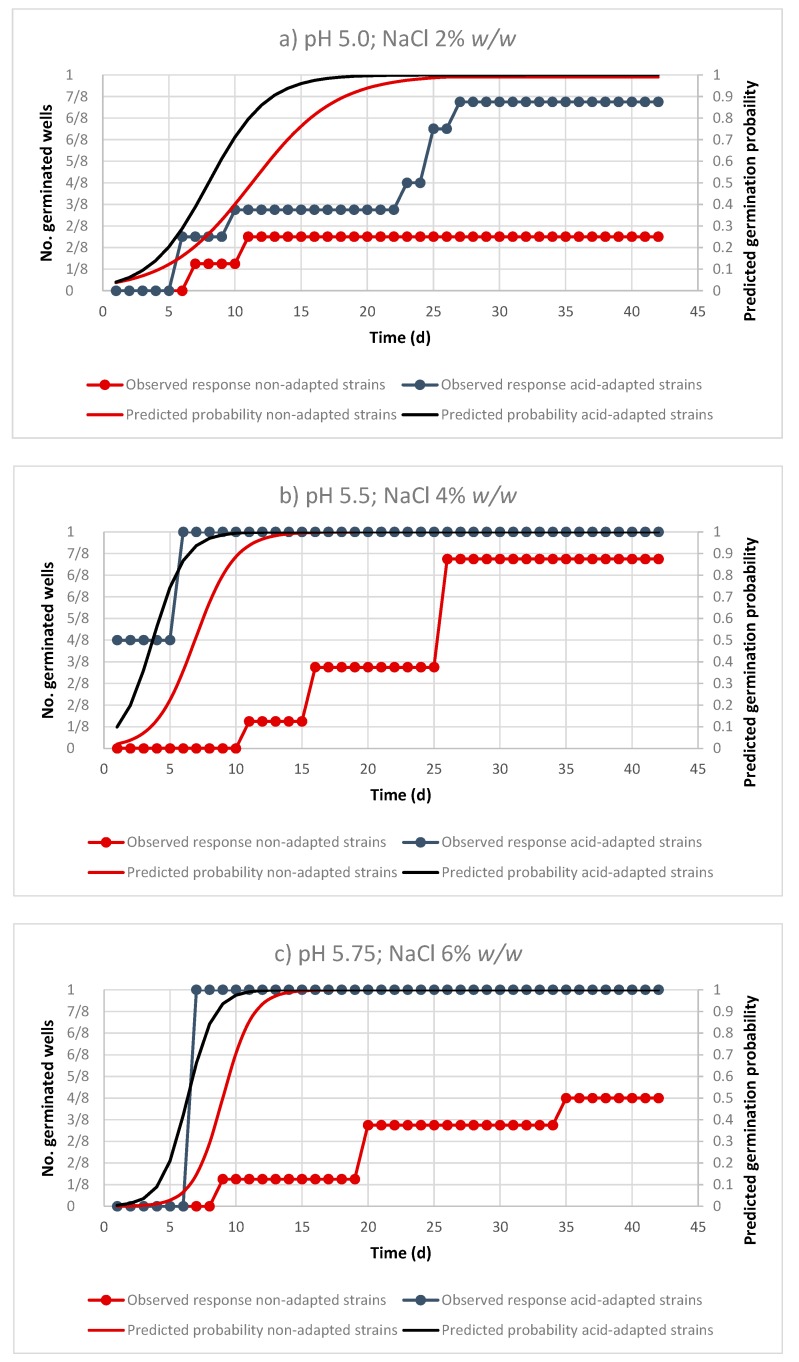
Evolution of predicted probabilities of germination and observed responses over experimental time for the non-adapted and acid-adapted strains of *C. sporogenes* at different combinations of pH and NaCl (panels **a**–**c**).

The evolution of germination probabilities in comparison with the observed responses at representative pH and NaCl conditions are shown in Figure 3. Overall, predictions given by the logistic models indicated earlier germination for the acid-adapted strains at all assayed conditions. At pH 5.0 and 2% NaCl, the logistic model predicted germination (*p* ≥ 0.125) after 5.08 and 3.73 days for the non-adapted and acid-adapted strains, respectively. The observations indicated that 7 out of the 8 wells showed germination for the acid-adapted strains, while the probability was reduced to two out of eight germinated wells for the non-adapted strains at the end of the incubation period (Figure 3a). When increasing pH and NaCl concentrations (Ph 5.5, 4% NaCl, and pH 5.75, 6% NaCl), germination responses occurred in a shorter period, since all wells of the acid-adapted strains led to positive germination after 7 days’ incubation (Figure 3b,c). On the contrary, the increase in NaCl concentration gave fewer germinated wells in the case of non-adapted strains (7 and 3 out of 8 wells, respectively). The incubation times predicted by the model for the germination of acid-adapted and non-adapted strains predicted were 1.33 and 3.95 days (pH 5.5 and NaCl 4%), and 4.38 and 6.8 days (pH 5.75, NaCl 6%), respectively.

### 3.3. Germination of C. sporogenes Strains in Table Olive Brines

Validation at different pH and NaCl levels was also performed in formulated brines anaerobically stored at 30 °C for 13 d. Overall, 208 conditions for the non-adapted and the acid adapted *C. sporogenes* strains were assessed. Regarding the non-adapted strains, non-germination and germination responses of *C. sporogenes* were produced in 59 and 45 conditions, respectively. The logistic regression model was able to correctly predict microbial evolution in 87.5% of the cases, though there were 10 fail-safe (9.61%) conditions (germination was predicted by the model, but not observed) and three fail-dangerous (2.88%) (no germination predicted, but observed). The results agreed with those obtained in DRCM, since germination was observed at pH 5.0 and 2% Knack, as well as at pH 5.5 and 4% NaCl. On the contrary, when NaCl increased up to 6%, germination was only observed at pH 6.0. 

For the acid-adapted strains, the logistic model was able to predict 81.73% of cases, results that were more conservative than those provided for the non-adapted strains, as confirmed by the increased number of fail-safe conditions (17, 16.34%). However, only two conditions were classified as fail-dangerous (1.92%). Such behavior can be explained by the more difficult germination of *C. sporogenes* in brines than in DRCM. Nevertheless, acid-adapted *C. sporogenes* strains were able to germinate at pH 5.0 and 4% Knack, as well as at pH 5.5 and 6% NaCl, thus confirming their higher resistance to stringent conditions when compared with the non-adapted strains.

For assessing the model’s application, the predicted time required for germination (Pred_t_model_) was calculated, at a probability of 0.125, at the studied conditions, and their results compared with those observed in brines (Obs_t_brine_) (Table 6). Predictions were conservative in most cases and provided a reasonable estimation of the germination time at different pH and NaCl conditions. Therefore, the presence of antimicrobial compounds in brines (organic acids, polyphenols, etc.) may have limited the germination of the acid-adapted *C. sporogenes* strains in table olive brines with respect to that observed in DRCM. Further studies are needed to confirm the effect of environmental factors and preservatives on the germination ability and microbial resistance of spore-forming bacteria in brines.

## 4. Discussion

In the present study, the acid-adaptation of *C. sporogenes* strains have influenced the subsequent germination responses as a function of different pH and NaCl conditions. Overall, acid-adapted spores produced faster germination at more limiting conditions when compared to the non-adapted ones. There are several studies in literature dealing with *C. sporogenes* behavior against various environmental conditions in different culture media using non-acid-adapted cells [18,35], as well as in food matrices such as meat products [2,16,20,31] or dairy [19,36]. However, although the growth ability of *C. botulinum* (or *C. sporogenes* as a surrogate) at low pH has been extensively reported, there are very few studies dealing with the effect of acid adaptation. 

Crosthwait [37] found that acid adaptation of *C. sporogenes* in FTM and tomato serum produced germination at lower pH values (4.85) than those initially observed without any adaptation (5.4). However, it was observed that adaptation ability was maintained by continuously sub-culturing at pH 5.0. In our study, *C. sporogenes* could germinate at pH 5.0, while no germination was observed at pH 4.5 during the 42-days incubation period. Lund et al. [21] reported minimum values for pH of 4.6 to produce the growth of vegetative strains of proteolytic *C. botulinum*, though this effect was time- and strain-dependent. Other authors have confirmed these results, such as Wong et al. [24], who found spore germination and outgrowth in anaerobically-acidified media at pH < 4.6. The effect of acid pH upon the germination and subsequent growth of *C. sporogenes* or *C. botulinum* strains in culture media is variable depending on several factors, such as the inoculum size, the redox potential or the presence of antimicrobial preservatives [18,30]. It is also recognized that the physiological state and properties of spores may vary between different batches of the same strains, thus increasing the variability of the probability of germination at acidic pH. 

Besides, it is reported that the addition of NaCl at high levels delays the germination and outgrowth of Clostridial strains. The relative effect of NaCl on the inhibition of *Clostridium* sp. may differ according to other factors that produce a synergistic effect or have higher significance than NaCl itself [18]. Whiting and Call [38] found that the time to the growth of proteolytic *C. botulinum* was delayed at temperatures <20 °C and pH levels <5.5, having NaCl no or little effect at concentrations ≤3%. However, when NaCl is added to food matrices, the inhibitory effect is usually enhanced. Taylor et al. [36] found that NaCl at 1.6% or 2.4% produced inhibition on *C. sporogenes* in canned butter samples. The same conclusion about the effect of NaCl was found by Knanipour et al. [19] in high moisture cheese. This result can be attributed by the effect of added food preservatives or the physical properties of foods, which can interfere with the growth of *Clostridium* sp.

Our results have confirmed previous findings in which pH and NaCl combinations could delay or inhibit the germination of spores. Montville [22] described the interaction of pH and NaCl on the growth of *C. botulinum*, reporting that germination was produced at pH 5.0 in the absence of NaCl, while concentrations up to 6% inhibited it at all of the pH levels tested. However, according to our results, the acid-adapted spores germinated faster at pH 5.0 than the non-adapted cells at NaCl concentrations of 0% and 2% (Figure 2a,b). The germination responses of acid-adapted cells were more marked at pH 5.5 when NaCl concentration ranged between 2% and 4% (Figure 2c,d), as well as at pH 5.75 and increased levels of NaCl (4% and 6%) (Figure 2e,f). The inhibitory effect of acid conditions is usually linked to the undissociated form of the acid, which dissociates into H+ and the anion in the bacterial cell. The increased concentration of protons causes a decrease in the intracellular pH, thus, disrupting cell metabolism. It is plausible that the interaction between increased NaCl concentrations and acidic pH could contribute to the increase of the turgor pressure of the cell, which in turn, may delay or prevent the germination of non-adapted spores [39]. Zhao et al. [30] reported that proteolytic *C. botulinum* did not grow at pH values < 5.5 and NaCl concentrations > 4% in a 14-day incubation period. These results match with those found in our study, since no germination was observed in 10 d at pH 5.5 and NaCl ≥ 4% for non-adapted strains and pH 5.5 and 6% NaCl for the acid-adapted ones. Sensitivity to the pH of *Clostridium* strains can produce a shift in the inhibitory pH–NaCl combinations. Montville [22] found that 6% NaCl at pH 5.5 inhibited the growth of proteolytic *C. botulinum* with intermediate pH sensitivity. Likewise, Graham et al. [40] did not obtain growth at pH less than 5.1 or 5% NaCl for non-proteolytic *Clostridium* strains. However, in our study, germination was produced at 6% NaCl and pH 5.0 for both non-adapted and acid-adapted strains (Figure 3a,b). Potential inter-strain specific differences and the use of a strain-cocktail may explain the variability in the environmental conditions allowing germination. 

The effect of oxygen concentration on *Clostridium* sp. growth has been recently studied by Couvert et al. [41], finding that total inhibition for *C. sporogenes* growth is reached at the 3.26% oxygen level in the gaseous phase. Nevertheless, when other conditions are suboptimal, much lower concentrations of oxygen and lower redox potentials may be inhibitory. 

The effect of the redox potential of the culture media in the presence of acid-adapted spores of *Clostridium* sp. is a matter of research for further studies, since it would allow a better understanding of the microbial behavior under suboptimal conditions.

Table 7 presents a comparison table of the observed growth responses of Clostridial strains published in earlier studies, with predicted germination probabilities found by the logistic regression models developed in this study. Though microbial responses were highly variable depending on the observation time, the strains used and the NaCl and pH combinations, predicted germination probabilities were higher for the acid-adapted strains when the outcome (*p*) was between 0 and 1.

The model’s predictions were mostly in agreement with the reported responses, since more than 68% of conditions have been corrected, classified by the models. Additionally, there was a 25% of conditions (8 out of 32) classified as fail-safe by the non-adapted and acid-adapted logistic regression models of *C. sporogenes*; i.e., no germination was predicted while growth was observed for non-adapted *C. sporogenes* strains. The fail-safe predictions obtained could probably be attributed to the variability in microbial behavior against the studied environmental factors, or the physiological differences of the strains used. Inoculation level (10^6^ spores/well) used in the present study may influence on the location of the germination boundary which is experimentally found at more limiting conditions when the inoculum size is large [42,43]. However, as many studies have pointed out, it is necessary to employ high inoculation levels to know the extent of a preservation system in a specific food under foreseeable conditions likely to occur in practice [18]. As the inoculation level usually used in these cases may exceed the actual contamination that could occur in food, the models’ predictions tend to be fail-safe. If germination is not observed under certain combinations of factors using such inoculum size, the implementation of such formulations in foods remains safe, since the germination probability will be unlikely. However, bias to the fail-safe is more preferred than for the fail-dangerous zone, since the model can provide conservative formulations of pH and NaCl for food operators. Finally, the percentages of fail-dangerous predictions were 6.25% and 3.12% for the non-adapted and acid-adapted logistic regression models of *C. sporogenes* i.e., germination was predicted while no growth was observed.

Overall, it should be remarked that the predictions provided in Table 7 may be taken with caution, since as described above, the comparison with external literature data is subjected to different variability sources that could not be considered by the logistic regression models here developed. Further, most of these studies are referring to growth kinetics of *C. sporogenes* in different matrices, and not to germination probability, so that the comparison with our results can be limited.

The results shown in the present study could have important implications in low-acid, fermented vegetables such as table olives, in which *Clostridium* sp. may not be present during their shelf-life [44]. However, the risk of cross-contamination and its survival increase in some elaborations such as black ripe table olives (Californian style), in the case of an insufficient heat treatment due to their high pH packaging levels (>6.0), or in green table olives with a reduced NaCl content [14]. Also, some specialities, such as Aloreña de Málaga table olives, might be exposed to similar risk when, to prevent the transformation of chlorophylls to pheophytins (loss of freshness, favored in acidic medium [45]), the packaging pH levels are set close to the *Clostridium* sp growth limits. Besides, dressing such as herbs or spices are vehicles of contamination of the *Clostridium* sp. in the final product [46]. 

Anaerobic fermentation may produce the outgrowth and toxin production of *Clostridium* sp., but also this could happen in microaerophilic environments given the tolerance of this microorganism to low-oxygen concentrations [41]. It is widely reported that a constant monitoring of pH < 4.6 guarantees the inhibition of *Clostridium* sp. in table olives. However, as above mentioned, *C. botulinum* was reported to survive and grow at this pH level in culture media. Thus, the risk of toxin production is not negligible.

Besides, the present study demonstrated that the adaptation of strains to acidic pH produces faster germination at moderate pH (5.0–5.75) and NaCl concentrations (4%–6%) in comparison to non-adapted cells. Although no germination was observed at pH 4.5, it could be plausible that acid-adapted cells could survive at this pH and produce germination at shorter incubation periods than non-adapted ones. Further research is needed to elucidate the metabolic pathways involved in acid adaptation and the subsequent germination of *Clostridium* strains.

In summary, the logistic models developed in this study successfully describe the observed data and quantify the effect of pH, NaCl and incubation time on the probability of germination of *C. sporogenes* in a laboratory medium, with good prediction results in natural green olive brines. This study provides the first guidance to food operators and the table olive industry on the selection of alternative formulations, although further studies should be carried out to validate these results under real table olive fermentation/packaging.

## Figures and Tables

**Table 1:** Experimental design for the selection of training (gray) and validation (white) conditions for the development of the logistic regression models (experimental time from 1 to 42 days).

	pH							
NaCl (%, *w*/*w*)	4.00	4.50	5.00	5.50	5.75	6.00	6.50	7.00
0								
2								
4								
6								

**Table 2:** Parameter estimates of the logistic regression models for the acid-adapted and non-adapted *C. sporogenes* strains.

	Coefficient	Estimate	S.E.	Wald	df	*P*-Value	Lower C.I (95%)	Upper C.I (95%)
**Acid-Adapted *C. sporogenes* Strains**	*time*	−3.175	0.527	36.289	1	<0.001	−4.208	−2.142
	*pH*	20.898	8.639	5.851	1	0.016	3.965	37.831
	*NaCl*	1.291	0.410	9.903	1	0.002	0.487	2.095
	*Time* × *pH*	0.726	0.113	41.182	1	<0.001	0.504	0.948
	*pH^2^*	−1.616	0.714	5.126	1	0.024	−3.016	−0.217
	*NaCl^2^*	−0.328	0.075	19.331	1	<0.001	−0.474	−0.182
	*constant*	−68.996	25.954	7.067	1	0.008	−	−
**Non-Adapted *C. sporogenes* Strains**	*time*	−3.610	0.382	89.462	1	<0.001	−4.358	−2.862
	*NaCl*	−6.566	1.620	16.424	1	<0.001	−9.741	−3.390
	*Time* × *NaCl*	0.059	0.014	17.743	1	<0.001	0.032	0.087
	*NaCl^2^*	−0.343	0.062	30.180	1	<0.001	−0.466	−0.221
	*ln(pH)* × *time*	2.364	0.248	91.015	1	<0.001	1.878	2.850
	*ln(pH)* × *NaCl*	4.514	0.943	22.927	1	<0.001	2.666	6.362
	*constant*	−3.565	0.555	41.218	1	<0.001	−	−

**Table 3:** Goodness of fit statistics for the logistic regression models of the acid-adapted and non-adapted *C. sporogenes* strains.

Goodness of Fit/Predictive Power	Acid-Adapted *C. sporogenes* Strains	Non-Adapted *C. sporogenes* Strains
	Coefficient	Coefficient
−2lnL ^1^	119.848	195.89
AIC ^2^	133.848	209.89
Hosmer-Lemeshow (df = 8)	2.065	6.044
*p*-value	0.979	0.642
Nagelkerke R^2^	0.950	0.921

^1^ log-likelihood, ^2^ Akaike Information Criterion.

**Table 4:** Classification tables of observed vs. predicted conditions of the training and validation datasets for the acid-adapted and non-adapted *C. sporogenes* strains.

ACID-ADAPTED *C. SPOROGENES* STRAINS				
Training	Estimated Probability		Total	Correct Prediction (%)
Observed response	No germination	Germination		
No germination	292	32	324	90.12
Germination	1	683	684	99.85
Total	293	715	1008	96.83
Validation	Estimated Probability		Total	Correct Prediction (%)
Observed response	No germination	Germination		
No germination	92	12	104	88.46
Germination	0	232	232	100.00
Total	92	244	336	96.43
NON-ADAPTED C. SPOROGENES STRAINS				
Training	Estimated Probability		Total	Correct Prediction (%)
Observed response	No germination	Germination		
No germination	311	55	366	84.97
Germination	0	642	642	100.00
Total	311	697	1008	94.54
Validation	Estimated Probability		Total	Correct Prediction (%)
Observed response	No germination	Germination		
No germination	89	26	115	77.39
Germination	0	221	221	100.00
Total	89	247	336	92.26

**Table 5:** Environmental conditions where a binary response was observed for *C. sporogenes* strains for the model and validation datasets after 42 d incubation at 30 °C.

Non-Adapted Strains					Acid-Adapted Strains		
pH	NaCl (%)	Germination	Dataset	pH	NaCl (%)	Germination	Dataset
5.0	0.0	5/8	Training	5.0	0.0	6/8	Training
5.0	2.0	2/8	Validation	5.0	2.0	7/8	Validation
5.0	4.0	3/8	Training	5.0	4.0	5/8	Training
5.0	6.0	4/8	Training	5.0	6.0	7/8	Training
5.5	6.0	5/8	Training	5.5	0.0	7/8	Validation
5.75	0.0	5/8	Training				
5.75	4.0	5/8	Training				
5.75	6.0	4/8	Validation				

**Table 6:** Comparison between the observed time to germination in table olive brines (Obs_t_brine_) of non-adapted and acid adapted *C. sporogenes* strains and those predicted (Pred_t_model_) by the logistic models.

Non-Adapted *C. sporogenes* Strains					Adapted *C. sporogenes* Strains		
pH	NaCl (%)	Obs_t_brine_	Pred_t_model_	pH	NaCl (%)	Obs_t_brine_	Pred_t_model_
5.0	2	8	5.07	5.0	2	6	3.72
5.0	4	>13	9.96	5.0	4	10	6.70
5.0	6	>13	>13	5.0	6	>13	>13
5.5	2	3	1.37	5.5	2	2	1
5.5	4	8	3.93	5.5	4	1	1.34
5.5	6	>13	9.26	5.5	6	>13	6.18
6.0	4	1	1.18	6.0	4	1	1
6.0	6	3	4.93	6.0	6	3	3.30

**Table 7:** Reported growth responses of *C. botulinum* and *C. sporogenes* strains in different published studies performed in culture media and their comparison with predictions of the probability of germination of the non-adapted and acid-adapted *C. sporogenes* strains used in the present study.

Microorganisms	T (°C)	pH	NaCl (%)	Growth	Obs. Time (Days)	Reference	*p*^1^ (non-Adapted)	*p* (Acid-Adapted)
*C. botulinum* proteolytic	30	4.7	2.5	No	>42	FSA (UK) ^2^	Yes (0.97) (fs)	Yes (0.99) (fs)
*C. botulinum* proteolytic	30	4.8	1.5	Yes	8.96	FSA (UK)	Yes (0.13) (c)	Yes (0.13) (c)
*C. botulinum* proteolytic	30	5.6	5.5	Yes	>42	FSA (UK)	Yes (0.99) (c)	Yes (1.00) (c)
*C. botulinum* proteolytic	30	6.3	5.5	Yes	11.08	FSA (UK)	Yes (0.99) (c)	Yes (1.00) (c)
*C. botulinum* proteolytic	30	5.2	1.5	No	>42	FSA (UK)	Yes (0.99) (fs)	Yes (1.00) (fs)
*C. botulinum* proteolytic	30	5.3	4.5	Yes	12.96	FSA (UK)	Yes (0.83) (c)	Yes (0.98) (c)
*C. botulinum* proteolytic	30	5.1	3.5	Yes	11.00	FSA (UK)	Yes (0.48) (c)	Yes (0.86) (c)
*C. botulinum* proteolytic	30	7	5.5	Yes	21.17	FSA (UK)	Yes (1.00) (c)	Yes (1.00) (c)
*C. botulinum* proteolytic	25	4.7	0.5	Yes	11.88	FSA (UK)	No (0.07) (fs)	No (0.04) (fs)
*C. botulinum* proteolytic	25	5.9	3.5	Yes	8.31	FSA (UK)	Yes (0.97) (c)	Yes (0.99) (c)
*C. botulinum* proteolytic	25	5.7	4.5	Yes	14.05	FSA (UK)	Yes (0.99) (c)	Yes (0.99) (c)
*C. botulinum* proteolytic	25	5.6	5.5	Yes	29.02	FSA (UK)	Yes (1.00) (c)	Yes (1.00) (c)
*C. botulinum* proteolytic	25	7	4.5	Yes	8.31	FSA (UK)	Yes (1.00) (c)	Yes (1.00) (c)
*C. botulinum* proteolytic	35	4.7	0.5	No	>42	FSA (UK)	Yes (0.46) (fs)	Yes (0.98) (fs)
*C. botulinum* proteolytic ^3^	30	7	0.6	Yes	1.00	Juneja et al. 1999 ^4^	Yes (0.20) (c)	Yes (0.65) (c)
*C. sporogenes*	37	7	4	Yes	>42	*18*	Yes (1.00) (c)	Yes (1.00) (c)
*C. sporogenes*	37	5.5	4	Yes	>42	*18*	Yes (1.00) (c)	Yes (1.00) (c)
*C. sporogenes ^5^*	30	5.5	2	Yes	1.00	*16*	No (0.10) (fd)	Yes (0.30) (c)
*C. sporogenes ^5^*	29.6	5.6	6	Yes	1.00	*16*	No (0.00) (fd)	No (0.00) (fd)
*C. botulinum* proteolytic ^6^	30	6.8	2	Yes	14.00	*21*	Yes (1.00) (c)	Yes (1.00) (c)
*C. botulinum* proteolytic ^6^	30	5.1	2	Yes	14.00	*21*	Yes (0.78) (c)	Yes (0.96) (c)
*C. botulinum* proteolytic ^6^	30	4.9	2	Yes	14.00	*21*	Yes (0.56) (c)	Yes (0.84) (c)
*C. botulinum* proteolytic ^6^	30	4.9	2	No	14.00	*21*	Yes (0.41) (fs)	Yes (0.65) (fs)
*C. botulinum* proteolytic ^6^	30	4.8	2	No	14.00	*21*	Yes (0.26) (fs)	Yes (0.35) (fs)
*C. botulinum* proteolytic ^6^	30	4.7	2	No	14.00	*21*	No (0.07) (c)	No (0.10) (c)
*C. botulinum* proteolytic	30	5.5	4	No	>42	*30*	Yes (1.00) (fs)	Yes (1.00) (fs)
*C. botulinum* proteolytic	30	5.5	2	Yes	>42	*30*	Yes (1.00) (c)	Yes (1.00) (c)
*C. botulinum* proteolytic	30	6	4	Yes	>42	*30*	Yes (1.00) (c)	Yes (1.00) (c)
*C. botulinum* proteolytic ^7^	30	5.5	3	Yes	3	*23*	Yes (0.19) (c)	Yes (0.61) (c)
*C. botulinum* proteolytic	30	5.0	0	Yes	30	*22*	Yes (0.91) (c)	Yes (1.00) (c)
*C. botulinum* proteolytic	30	5.0	3	No	30	*22*	Yes (1.00) (fs)	Yes (1.00) (fs)
*C. botulinum* proteolytic	30	5.5	4	Yes	30	*22*	Yes (1.00) (c)	Yes (1.00) (c)
TOTAL (c/fs/fd) ^8^							68.75%/25%/6.25%	71.88%/25%/3.12%

^1^*p* (probability of germination estimated by the logistic regression models). ^2^ Food Standard Agency (UK). ^3^.Mixed strains culture of *C. botulinum* proteolytic: 62A 33 999 C11, Serotype(s): A A B B. ^4^ Juneja, V.K.; Whiting, R.C.; Marks, H.M.; Snyder, O.*p*. Predictive model for the growth of *Clostridium perfringens* at temperatures applicable to cooling of cooked meat. *Food Microbiol*. **1999,** *16*, 335-349. doi: 10.1128/AEM.70.5.2728-2733.2004. ^5^ Until the stationary phase was reached. Probability of germination was calculated at time 24 h. ^6^ Mixed strains culture of *C. botulinum* proteolytic. Type A strains ZK3, 62A, VL1, 16,037 and NCTC 3805, and proteolytic type B strains 2345, B6, NCIB 10657, 3262 and 3266. Vegetative bacteria were inoculated. ^7^ Mixed strains culture of *C. botulinum* proteolytic. Type A strains 17409, 62A, 25763; proteolytic type B strains 7949, 53B, and B-aphis (proteolytic type B), and type C: A028. ^8^ TOTAL is referred to the percentage of correct (c), fail-safe (fs) and fail-dangerous (fd) predictions classified by the non-adapted and acid-adapted logistic regression models of *C. sporogenes*.
